# Molecular Self-Assembly Strategy for Generating Catalytic Hybrid Polypeptides

**DOI:** 10.1371/journal.pone.0153700

**Published:** 2016-04-26

**Authors:** Yoshiaki Maeda, Justin Fang, Yasuhiro Ikezoe, Douglas H. Pike, Vikas Nanda, Hiroshi Matsui

**Affiliations:** 1 Department of Chemistry, Hunter College and the Graduate Center, City University of New York, New York, New York, United State of America; 2 Department of Biochemistry, Center for Advanced Biotechnology and Medicine and the Department of Biochemistry and Molecular Biology, Robert Wood Johnson Medical School, Rutgers, The State University of New Jersey, Piscataway, New Jersey, United State of America; 3 Department of Biochemistry, Weill Medical College of Cornell University, New York, New York, United State of America; Brandeis University, UNITED STATES

## Abstract

Recently, catalytic peptides were introduced that mimicked protease activities and showed promising selectivity of products even in organic solvents where protease cannot perform well. However, their catalytic efficiency was extremely low compared to natural enzyme counterparts presumably due to the lack of stable tertiary fold. We hypothesized that assembling these peptides along with simple hydrophobic pockets, mimicking enzyme active sites, could enhance the catalytic activity. Here we fused the sequence of catalytic peptide CP4, capable of protease and esterase-like activities, into a short amyloidogenic peptide fragment of Aβ. When the fused CP4-Aβ construct assembled into antiparallel β-sheets and amyloid fibrils, a 4.0-fold increase in the hydrolysis rate of *p*-nitrophenyl acetate (*p*-NPA) compared to neat CP4 peptide was observed. The enhanced catalytic activity of CP4-Aβ assembly could be explained both by pre-organization of a catalytically competent Ser-His-acid triad and hydrophobic stabilization of a bound substrate between the triad and *p*-NPA, indicating that a design strategy for self-assembled peptides is important to accomplish the desired functionality.

## Introduction

A number of polypeptides have been shown to promote protease-like reactions, utilizing specific folded conformations for controlling the catalytic activity.`^1^–^3^] While simple shorter oligopeptides have been demonstrated to show the significant catalytic activity and selectivity,[4, 5] intermediate length polypeptides (10–15 amino acids) generally do not lock in well-defined structures, limiting their catalytic performance.[1, 6, 7] Recently, there has been notable progress in developing polypeptides in this intermediate length range that carry out chemical reactions including hydrolysis and condensation as well as inorganic crystal growth by stabilizing the conformation for the catalysis with various electrostatic self-assembly strategies.[8–12]

In this work, we examined whether fusing a catalytic peptide to a short amyloidogenic peptide fragment could modulate catalytic activity by (1) stabilizing an active conformation, and (2) providing a hydrophobic surface for promoting substrate binding. This approach is aimed to constrain polypeptides in rigid structures akin to enzyme active sites. A CP4 polypeptide (SMESLSKTHHYR),[3] whose sequence was previously identified by the hydrogel-based phage library for promoting protease and esterase reactions, was used as a model system. Traditionally, phage display has been used to identify peptide binders to cells, inorganics, organics, and carbon materials,[13–19] however the hydrogel-based phage biopanning could discover the peptide sequences that promote chemical reactions. This polypeptide was fused with a fragment (FFKLVFF) of the amyloid-beta (Aβ) protein to generate the proposed hydrophobic antiparallel β–sheet pocket for binding substrates in higher affinity and selectivity.[20, 21] In addition to providing hydrophobicity, we hypothesized the Aβ peptide could drive macromolecular crowding of the peptide by forming amyloid fibrils.[22–24] Such crowding effects can entropically stabilize active conformations of otherwise dynamic peptides. For larger enzymes, immobilization and display on amyloid fibers can diminish catalytic activity by occluding substrate binding.[25] Due to their smaller size, this is expected to be less of an issue for catalytic peptides.

**Fig 1:**
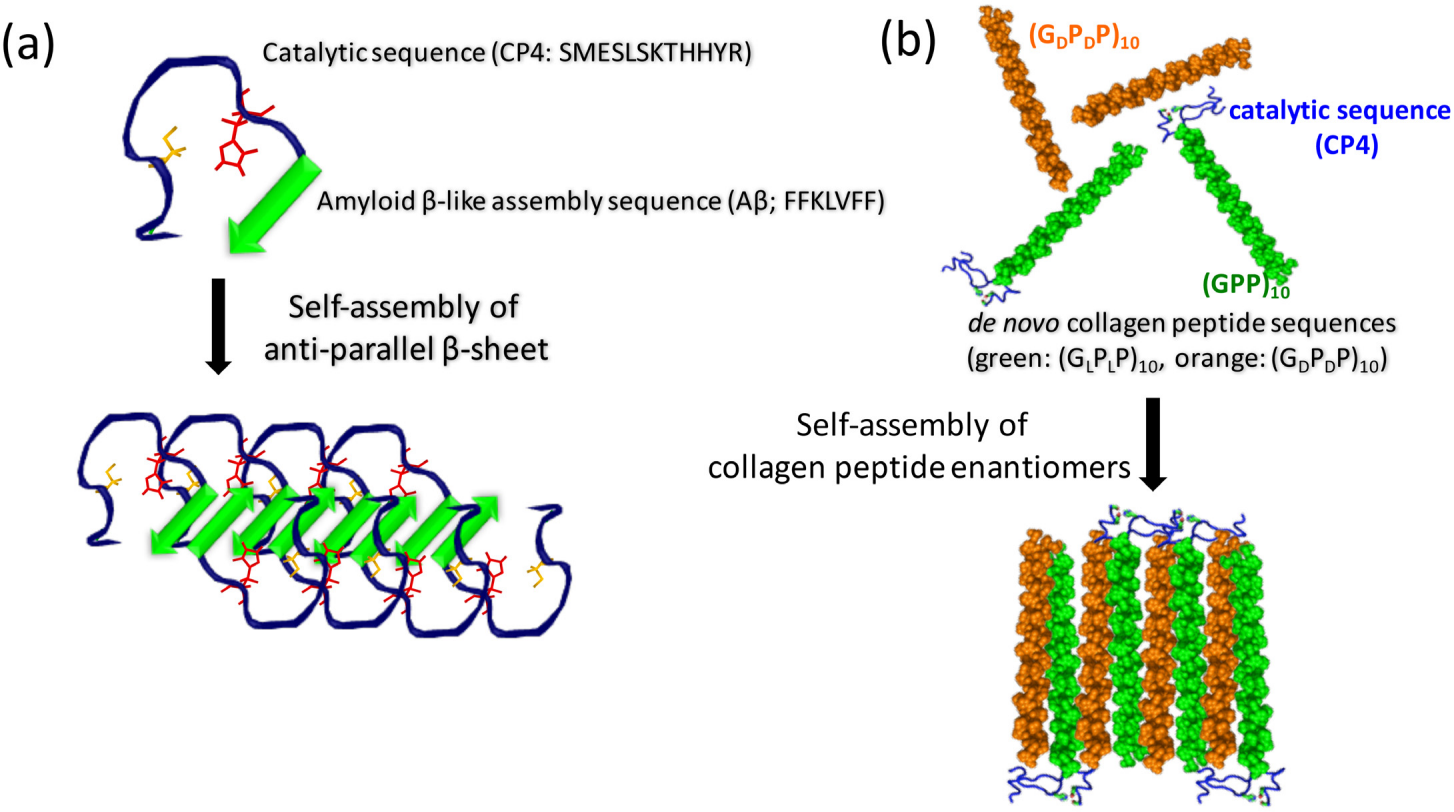
Schematic illustrations of the self-assembled structures of *de novo* hybrid peptides. (a) The self-assembly of *de novo* hybrid peptides that the sequence of CP4 catalytic peptide is fused with amyloid β peptide. The self-assembly of CP4-Aβ (SMESLSKTHHYRFFKLVFF) into 2D hydrophobic antiparallel β-sheet conformation increases the catalytic activity as compared to the non-assembled neat CP4 peptide. (b) Another sheet structure assembly by using *de novo* triple helix peptide as an assembly motif. The CP4 fused into (G_L_P_L_P)_10_ sequence can be assembled into the sheet *via* isometric association with (G_D_P_D_P)_10_.[40].

In this work we independently examined the effects of local hydrophobicity and crowding on CP4 activity (Fig 1). The Aβ fragment provides both crowding and a hydrophobic surface. In contrast, fusing CP4 to a designed self-assembling collagen mimetic peptide system provides crowding without the presence of adjacent hydrophobic groups. Since amyloid fibril conformation is known to be stable in harsh conditions such as organic solvents,[22, 23] there is a potential that this self-assembly approach could produce synthetic biocatalysts that function in environments inhospitable to natural enzymes.

## Materials and Methods

### Materials

All *de novo* peptides, CP4, Aβ, CP4-Aβ, (G_D_P_D_P)_10_, CP4-(G_L_P_L_P)_10_, and mutated CP4-Aβ peptides, were purchased from GenScript USA Inc. (Piscataway, NJ, USA) and used without any further purification. PBS solution (pH = 7.4) was prepared by dissolving a PBS tablet (Sigma-Aldrich) into ultrapure water, following the instruction from company. 10 mM phosphate buffer (pH = 7.0) was prepared by mixing 10 mM NaH_2_PO_4_ (Sigma-Aldrich) and 10 mM Na_2_HPO_4_ (Sigma-Aldrich).

### Ester hydrolysis analysis

The catalytic peptide solution, including CP4 or CP4-fused peptides such as CP4-Aβ or mutated CP4-Aβ peptides, was prepared by dissolving the peptide (1 mM) into PBS buffer (solution A). The substrate, *p*-nitrophenylacetate (*p*-NPA) was dissolved in dehydrated methanol (solution B, 25 mM). 440 μL of PBS was mixed with 50 μL of solution A and agitated by a vortex mixer (10 seconds), and 10 μL of solution B was subsequently added and agitated by a vortex mixer (10 seconds). The reaction time was set as zero at the time when the solution B was added to the mixture of solution A and PBS solution. In order to investigate the reaction kinetics, the absorbance at 400 nm was monitored using a UV/Vis spectrometer (Beckman Coulter, DU-800) at room temperature over time. Solutions including Aβ-fused peptide became cloudy due to the aggregation of the peptide, therefore, a reference solution, which was mixture of 440 μL of PBS, 50 μL of peptide solution, and 10 μL of methanol, was separately prepared to obtain a reference absorbance data. The reference data at each reaction time was subtracted from the absorbance of the reaction solution and the resulting data set was used to properly estimate the catalytic activity of CP4-Aβ. The calibration curve (data is not shown) of the product of the hydrolysis, *p*-nitrophenol (*p*-NP), was obtained by using 0 to 0.16 mM solution in PBS/MeOH (98:2 volume ratio), and the molar extinction coefficient of *p*-NP was estimated as 15600 (M^-1^cm^-1^). Catalytic activities of all peptides and their assemblies were calculated by initial reaction rates of kinetics curve except (4), whose activity was derived from the concentration of *p*-NP 1200 seconds after the peptide was mixed. Catalytic activities were normalized by the result of control experiment, *p*-NPA ester hydrolysis in PBS/MeOH without any catalytic peptides.

### Preparation of sheet-like self-assembly of collagen-mimicking triple helix peptide

To self-assemble the triple helices of CP4-(G_L_P_L_P)_10_ and (G_D_P_D_P)_10_ into the sheet structure, each peptide was self-assembled for 4 days at 4°C, monitored by CD spectrometer. These assemblies were then mixed and incubated for another 4 days at 4°C. 2 μL of peptide sample was placed on TEM grid and incubated for 10 min in Eppendorf tube (closed condition) at room temperature. 2 μL of water was added for washing, and after 30 seconds the water was dried with blotting paper (this process was repeated three times). 2 μL of 2% methylaminetungstate (pH = 6.7) was placed for negative staining and incubated for 1 min. 2 μL of water was added for the final washing, and after 30 seconds the water was removed with blotting paper and the grid was dried at room temperature.

### Generating Peptide Model Structures

Starting models for molecular dynamics simulations were constructed and geometrically optimized using the protCAD[26, 27] (protein Computer Automated Design) software platform. The protCAD software and relevant structure files in PDB format are included in an online repository (d). For monomeric CP4, the peptide sequence was mapped onto an extended chain followed by torsional relaxation of sidechain and backbone degrees of freedom. CP4-(GPP)_10_ was built by joining the CP4 N-terminal peptide in an extended conformation to the N-termini of the three chains of the high-resolution structure of a model collagen peptide (PDB ID 3B0S[28]), followed by torsional sidechain and backbone optimization. CP4-Aβ was constructed *de novo* to adopt an anti-parallel β-sheet. The amyloid core backbone conformation was set to (φ, ψ) = (–135°, 135°). Each of the three chains were aligned to the Z-axis and translated 5 Å along the X-axis away from neighboring chains to mimic the inter-strand spacing in an amyloid β-sheet. To form the antiparallel beta-sheet, 2 of the 3 chains were rotated 180 degrees along the X-axis. CP4 modeled in an extended conformation was then fused to the N-terminus of each chain and the complete structure was optimized by sampling torsional degrees of freedom.

### Molecular Dynamics Simulations

For each peptide system, AMBER explicit molecular dynamics was run at 300 K for a total of 800 ns using the ff99sb force field[27, 29] with a distance cutoff of 10 Å. Models were solvated in TIP3P water boxes, minimized in 1500 steps of steepest descent, followed by 1500 steps of gradient minimization. A temperature ramp from 0 K to 300 K was performed over 100 ps, followed by 400 ns of full molecular dynamics at 300 K for equilibration. The subsequent 400 ns production run was used for analysis for all models.

### Identifying Potential Catalytic Sites

Scripts[30] were developed in protCAD[26] to parse the MD trajectory and identify the occupancy of hydrolysis competent structures over 400 ns of explicit molecular dynamics in 20 ps frame steps. A hydrolysis competent structure was defined by a distance cutoff of 5 Å between a hydroxyl group oxygen (serine or threonine), a carboxylate oxygen (glutamate, aspartate or the C-terminus), and either of the histidine imidazole nitrogens (Fig A in S1 File).

## Results and Discussion

**Fig 2:**
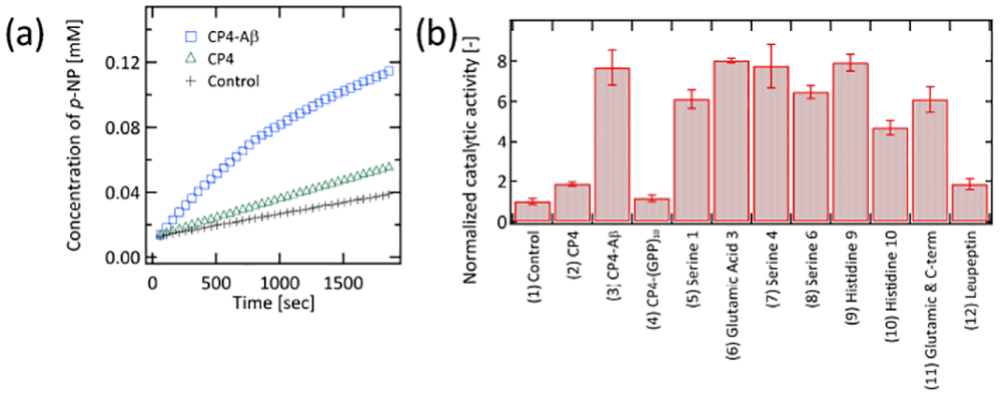
Structural analysis of the CP4-Aβ assembly. (a) TEM observation of nanofibers of the CP4-Aβ assembly. (b) UV/visible absorption of Congo red in the absence and presence of self-assembled CP4-Aβ peptides and Aβ peptides. (c) CD spectrometry of the CP4-Aβ peptide assembly in MeOH.

The ability of CP4-Aβ polypeptide (SMESLSKTHHYRFFKLVFF) to self-assemble into amyloid-like fibers was confirmed by transmission electron microscopy (TEM). The CP4-Aβ polypeptide (0.1 mM) was first dissolved in PBS/MeOH (98:2, volume ratio) mixed solution (pH7.4), and dropped on a TEM grid. After drying and washing with water, the sample was observed by TEM (JEM 2100, JEOL) under an acceleration voltage of 200 kV without staining. Since CP4-Aβ did not completely dissolve in PBS, the grid contained areas where monomers were aggregated and ones where the self-assembled domains were observed. On regions of self-assembly, the nanofiber structure, typically observed in the amyloid peptide assembly,[23] was clearly resolved, suggesting that the Aβ sequence could direct self-assembly of CP4-Aβ into nanofiber (Fig 2a). 15 nm diameter nanofibers and tape-like structures (30~40 nm in width) were both observed, consistent with the multiple assembling forms of the Aβ peptide.[23] In order to confirm the assembled structure of CP4-Aβ, Congo Red staining was performed (Fig 2b). 50 μM CP4-Aβ peptide was dissolved in PBS/MeOH (98:2, volume ratio) with 40 μM of Congo Red and incubated for 30 min at room temperature. Congo Red absorbance was greater and red-shifted in the presence of peptide, consistent with the amyloid-type β-sheet conformation.[23] It should be noted that a control of the neat Aβ peptide was also shown to be the same β-sheet structure. For further structural analysis, the self-assembly of CP4-Aβ was examined by circular dichroism (CD) spectroscopy (Fig 2c). The CD spectrum of CP4-Aβ shows a characteristic negative peak at 212 nm, supporting the Congo Red experiment that the CP4-Aβ peptide are assembled into the β-sheet conformation.[31] It should be noted that this CD spectrum also contains the characteristic peaks of α-helix from CP4 peptide (Fig B in S1 File).

**Fig 3:**
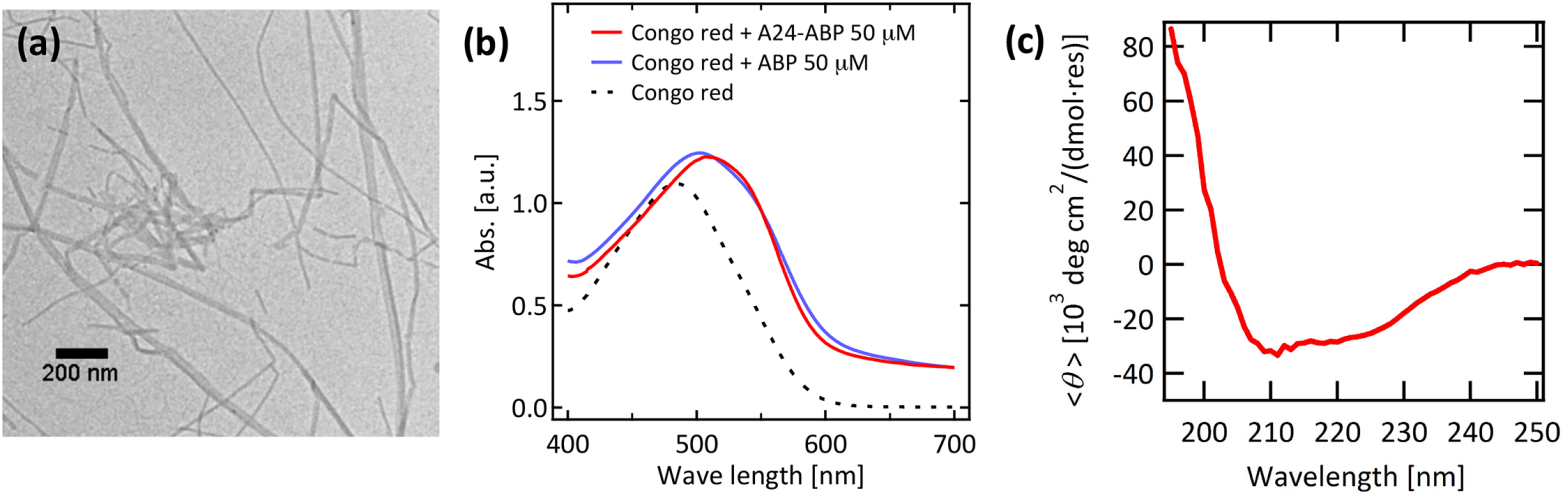
Kinetic studies of *p*-NPA ester hydrolysis catalyzed by the CP4-Aβ catalytic peptide assembly and the CP4 catalytic peptide monomers. (a) Monitoring the hydrolysis product of *p*-NP catalyzed by CP4-Aβ catalytic peptides with and without the self-assembly. (b) Normalized catalytic activities of CP4 peptides for *p*-NP generation with β-sheet self-assembly (2), without β-sheet self-assembly (3), with triple helical sheet self-assembly (4), and assembled ones where triads were mutated to alanine. (5)–(11) show the mutated position of CP4 peptide (SMESLSKTHHYRFFKLVFF) conjugated with the Aβ motif. (12) shows that the enzyme inhibitor of leupeptin reduces the activity of CP4-Aβ mimicking protease. Catalytic activities were derived from initial reaction rates (raw data were shown in Fig D in S1 File) and normalized by the result of control experiment, *p*-NPA ester hydrolysis in PBS/MeOH without any catalytic peptides.

To evaluate relative catalytic activities of various peptide constructs, *p*-nitrophenylacetate (*p*-NPA) hydrolysis was monitored with the CP4-Aβ assembly and non-assembled CP4 polypeptides, respectively. Absorbance of the product, *p*-nitrophenol (*p*-NP), at 400 nm was monitored in the absence of polypeptides as a standard to evaluate baseline rates of *p*-NPA hydrolysis. Absorbance was measured using a UV-Vis absorptiometer (Beckman Coulter, DU800), and concentration of *p*-NP was estimated using an appropriate calibration curve. Rates of *p*-NPA hydrolysis were compared with respect to the standard in PBS/MeOH (98:2, volume ratio) containing *p*-NPA (0.5 mM) and peptides (0.1 mM). The hydrolysis reaction proceeded much faster with the CP4-Aβ assembly than the CP4 monomer (Fig 3a). In addition, the higher initial velocity of turnover of CP4-Aβ assembly resembled the burst phase kinetics of hydrolyzing enzymes such as chymotrypsin,[32] while no initial burst phase was observed for CP4. The hydrolysis rate of CP4-Aβ assembly was estimated to be 7.7-fold as compared to the standard, while the CP4 polypeptide monomer was 1.9-fold (Fig 3b). Therefore, the fusion of the CP4 sequence into the Aβ fragment improved the catalytic performance in 4.0-fold, indicating that the catalytic performance of polypeptides can be improved significantly when these polypeptides were assembled into the amyloid-type β-sheet. While the enhancement of activity was accomplished by the molecular self-assembly strategy, the catalytic profile did not suggest Michaelis− Menten kinetics, rather a close to linear relationship within the concentration range studied, indicating that the K_m_ value is not yet approached under these conditions and therefore a k_cat_ value cannot be determined. This result implies a minor role of substrate binding in the catalysis.

One of the reasons that the CP4 polypeptide in the assembled structure is capable of promote the ester hydrolysis could be due to the amino acid sequence and their folded conformations. Interestingly, the CP4 polypeptide contains S/H/E, which are known to form a conventional triad catalytic center in natural protease.[33] *p*-NPA hydrolysis can be catalyzed by serine or histidine nucleophiles [34], and this assay has been used to assess the activity of natural proteases [32] and *de novo* designed catalysts.[35, 36] To obtain more insights into the reaction mechanism, we performed a series of mutation experiments where acids (glutamic acid E3 and C-terminus), serine (S1, S4, and S6), and histidine (H9 and H10) were substituted with alanine, respectively (C-terminus was amidated). As shown in Fig 3b, S1, S6, H10, and C-terminus are essential for the catalytic activity. We hypothesized that the drop of activity with S1 and S6 is smaller than other mutations because these serine residues are in close proximity; in the self-assembled structure, they can compensate each other in intermolecular hydrogen bonding as one of them is missing. Assuming this polypeptide assembly mimics protease, we also introduce the enzyme inhibitor of leupeptin, known to effectively inhibit serine proteases,[37] which indeed lowered the activity to 24% ((12) in Fig 3b). This observation supports our hypothesis that the significant drop of activity is induced as leupeptin inhibits all serine protease activities in the peptide. This result indicates that S/H/E triad plays an important role for the protease-like catalytic function and an acid group of C-terminus can also supplement glutamic acid in the activity.

**Fig 4:**
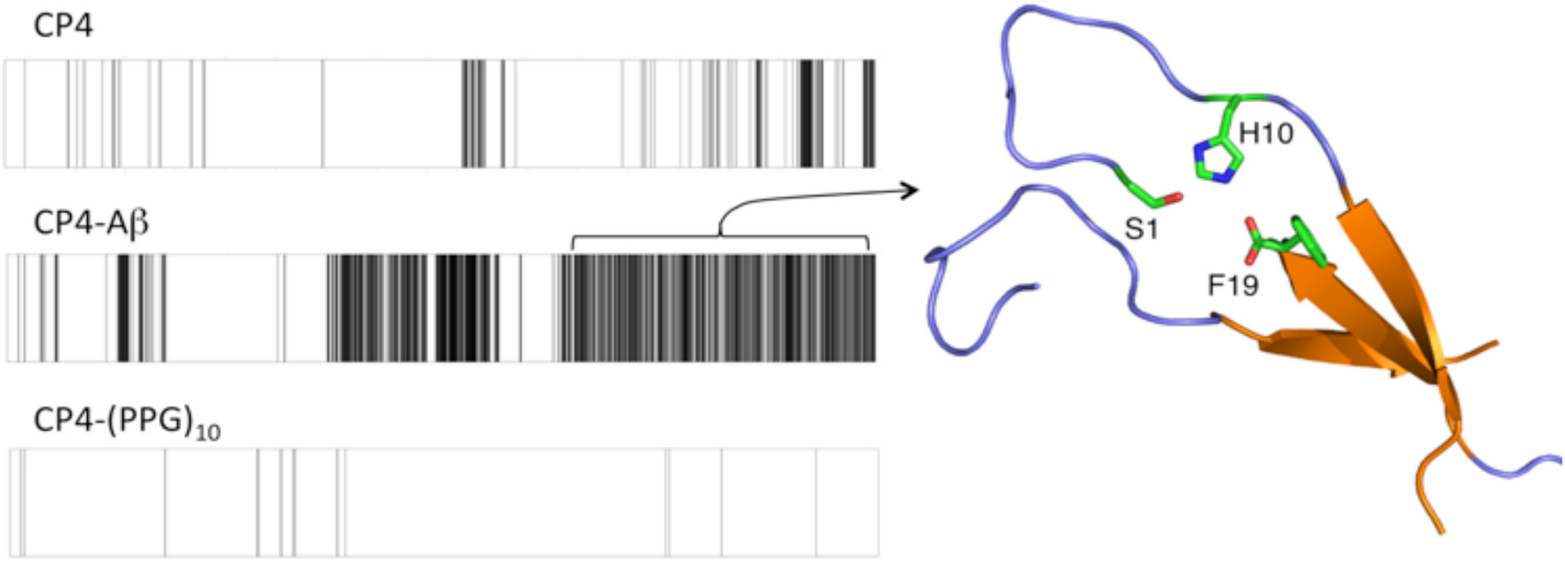
Analysis of molecular dynamics trajectories revealed low occupancy of hydrolysis-competent active sites in CP4 either alone or fused to the collagen triple-helix forming sequence (GPP)_10_. Black lines indicate instance of one or more clusters of H + S + acid groups in proximity to form a catalytically competent triad over 400 ns of all-atom explicit solvent dynamics. In contrast, CP4-Aβ modeled as three strands in an antiparallel beta-sheet shows significant triad occupancy. The most frequent triad observed in CP4-Aβ shows interactions between S1 and H10 on one chain and the F19 carboxy terminus of the amyloid domain of the adjacent chain.

To model the structural implications of amyloid formation on CP4 catalysis, detailed all-atom simulations were conducted on CP4 alone and fused to Aβ. A three-stranded anti-parallel β-sheet assembly of CP4-Aβ was optimized in *protCAD[26]* to approximate a minimal unit of the extended amyloid fibril. Using this model as a starting point, the polypeptide was equilibrated in explicit solvent and subject to 400 ns of molecular dynamics using the AMBER platform [38]. A geometric definition of triad consisting of S, H, and an acid group of E/C-terminus was used to identify the occupancy of catalytically competent sites during the course of the simulation trajectory (Fig 4). Consistent with the experimental studies, CP4-Aβ showed a significantly higher propensity to form incipient triads when compared to CP4 alone. Conformational clustering of the CP4-Aβ trajectory indicated the most common triad was formed by S1, H10 and the C-terminus of the amyloid sequence. These are the three positions that are the most sensitive to mutation or modification. The C-terminal F19 is ideally situated to stabilize the *p*-NPA substrate at this site through π-stacking interactions. Thus, the enhanced catalytic activity of CP4-Aβ could be explained both by pre-organization of a catalytically competent triad due to intermolecular interactions and hydrophobic stabilization of a bound substrate.

Molecular crowding at surfaces or in cells is known to enhance protein stability and modulate catalytic activity [39]. To explore whether a generic crowding mechanism could explain the improved catalytic activity of CP4-Aβ assembly, we examined another type of self-assembly of the hybrid polypeptide, the CP4 sequence fused to a *de novo* triple helix peptide sequence of (G_L_P_L_P)_10_ (Fig 1b). In this approach, the triple-helix would bring three CP4 chains in close proximity as a triple helix motif induces the self-assembly into nanoscale sheets upon association with the polypeptide stereoisomer (G_D_P_D_P)_10_ (Fig C in S1 File).[40] In this construct, crowding was expected but the assembly scaffold would not present a hydrophobic surface. The ester hydrolysis rate of CP4 was not improved as compare to the neat CP4 (Fig 3b). Consistent with this observation, very little formation of catalytic triad was shown in molecular dynamics modeling of the CP4-(GPP)_10_ fusion triple-helix (Fig 4). Thus, these results suggest that the amyloid-driven self-assembly of CP4 polypeptide provides a specific structural niche of the folded polypeptide chain that facilitates *p*-NPA hydrolysis for CP4.

In conclusion, the catalytic activity of CP4 polypeptides was improved by assembling them into hydrophobic β sheets when the CP4 sequence was fused to the amyloid sequence of Aβ. This CP4-Aβ hybrid polypeptide was assembled into amyloid-like nanofibers, and the catalytic activity was increased 4.0-fold as compared to the CP4. The self-assembly strategy helps improve the catalytic activity of polypeptides by crowding them and forming hydrophobic binding sites, however the outcome suggests that the conformation of polypeptide in the assembly should be taken into consideration. Although the enhancement is modest, molecular simulations indicate specific structural mechanisms through which the enhancement is achieved, providing opportunities for further enhancement by structure-guided protein design.

## Supporting Information

S1 File Fig A in S1 File. An example of an incipient S-H-acid catalytic triad in peptide MD trajectory. Fig B in S1 File. CD spectra of CP4 peptide and amyloidogenic peptide fragment of Aβ. A green line shows CP4 spectrum and a blue line shows Aβ peptide spectrum. CD spectra were obtained in methanol using AVIV CD instrument (Aviv Biomedical, Inc., Lakewood, NJ, US), and normalized to molar ellipticity. Fig C in S1 File. TEM image of self assembly of CP4-fused to a *de novo* triple helix peptides. (a) The triple helix sequence of (G_L_P_L_P)_10_, where CP4 was fused, was assembled with the peptide stereoisomer (G_D_P_D_P)_10_ to form the sheet-like structure. This structure is consistent with the one assembled from (G_L_P_L_P)_10_ and (G_D_P_D_P)_10_ without CP4. (b) TEM image of self assembly of the *de novo* triple helix peptides without the CP4 sequence. Fig D in S1 File. Raw data for Fig 3(b).
(PDF)
