# Supplementary material for: Molecular Self-Assembly Strategy for Generating Catalytic Hybrid Polypeptides
Source: PLoS One. 2016 Apr 26;11(4):e0153700. doi: 10.1371/journal.pone.0153700 (PMC4846159; doi:10.1371/journal.pone.0153700)
Supplement: S1 File — Fig A in S1 File. An example of an incipient S-H-acid catalytic triad in peptide MD trajectory. Fig B in S1 File. CD spectra of CP4 peptide and amyloidogenic peptide fragment of Aβ. A green line shows CP4 spectrum and a blue line shows Aβ peptide spectrum. CD spectra were obtained in methanol using AVIV CD instrument (Aviv Biomedical, Inc., Lakewood, NJ, US), and normalized to molar ellipticity. Fig C in S1 File. TEM image of self assembly of CP4-fused to a de novo triple helix peptides. (a) The triple helix sequence of (GLPLP)10, where CP4 was fused, was assembled with the peptide stereoisomer (GDPDP)10 to form the sheet-like structure. This structure is consistent with the one assembled from (GLPLP)10 and (GDPDP)10 without CP4. (b) TEM image of self assembly of the de novo triple helix peptides without the CP4 sequence. Fig D in S1 File. Raw data for Fig 3(b). (PDF) [file pone.0153700.s001.pdf]

# Supporting Information:

Molecular Self-Assembly Strategy for Generating Catalytic Hybrid Polypeptides

*Yoshiaki Maeda,*[a, #] *Justin Fang,*[a b, #] *Yasuhiro Ikezoe,*[a] *Douglas H. Pike,*[c] *Vikas Nanda,*[c,*] *Hiroshi Matsui*[a, b, d, *]

[a] *Department of Chemistry, Hunter College of the City University of New York, New York, NY 10065 (U.S.A.).*

[b] *Department of Chemistry, The Graduate Center of the City University of New York, New York, NY 10016 (U.S.A.).*

[c] *Department of Bicochemistry, Center for Advanced Biotechnology and Medicine and the Department of Biochemistry and Molecular Biology, Robert Wood Johnson Medical School, Rutgers, The State University of New Jersey, Piscataway, NJ 08854 (U.S.A).*

[d] *Department of Biochemistry, Weill Medical College of Cornell University , 413 E69th street, New York, NY 10021 (U.S.A).*

** to whom correspondence should be sent. E-mail: nanda@cabm.rutgers.edu, hmatsui@hunter.cuny.edu*

[#] these authors contributed equally to this work.

## Supplementary Figures.

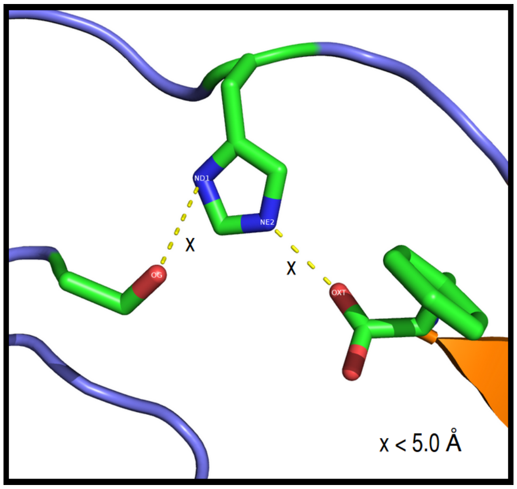


Figure A.  An example of an incipient Ser-His-acid catalytic triad in peptide MD trajectory.

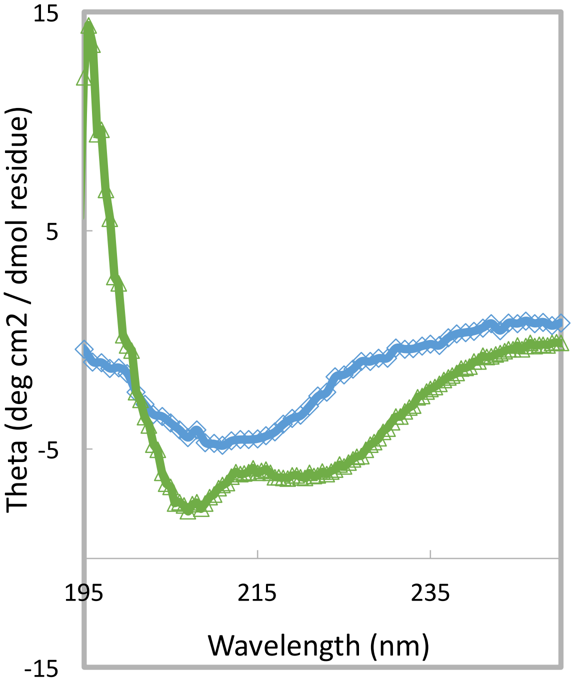


Figure B. CD spectra of CP4 peptide and amyloidogenic peptide fragment of Aβ. A green line shows CP4 spectrum and a blue line shows Aβ peptide spectrum. CD spectra were obtained in methanol using AVIV CD instrument (Aviv Biomedical, Inc., Lakewood, NJ, US), and normalized to molar ellipticity.

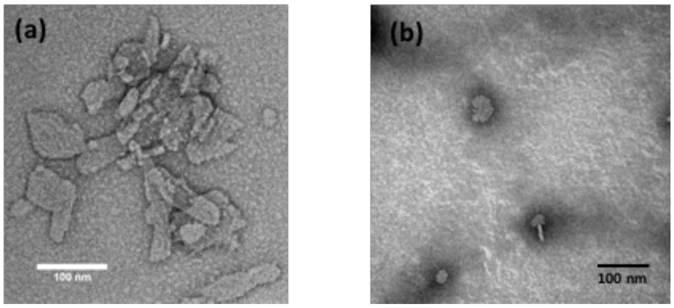


Figure C. TEM image of self assembly of CP4-fused to a *de novo* triple helix peptides. (a) The triple helix sequence of $(G_LP_LP)_{10}$, where CP4 was fused, was assembled with the peptide stereoisomer $(G_DP_DP)_{10}$ to form the sheet-like structure. This structure is consistent with the one assembled from $(G_LP_LP)_{10}$ and $(G_DP_DP)_{10}$ without CP4. (b) TEM image of self assembly of the *de novo* triple helix peptides without the CP4 sequence.

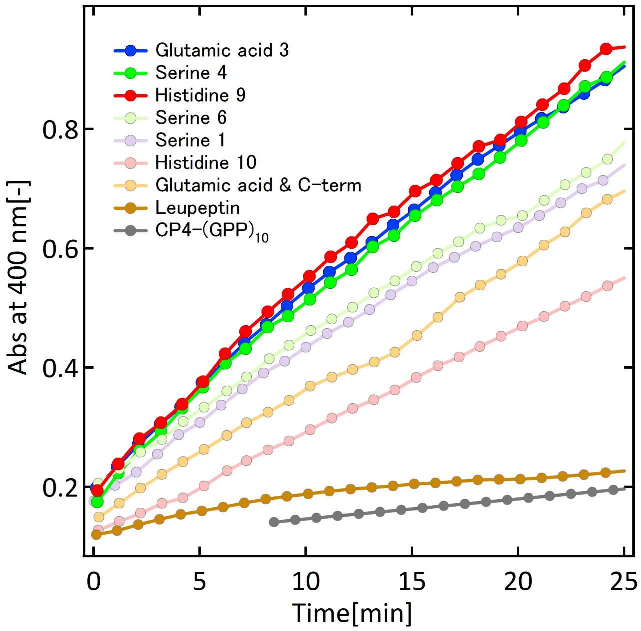


Figure D. Raw data for Figure 3-b.
